# Supplementary figures and images for: Monodisperse Fluorescent Polystyrene Microspheres for Staphylococcus aureus Aerosol Simulation
Source: Polymers (Basel). 2023 Aug 31;15(17):3614. doi: 10.3390/polym15173614 (PMC10490235; doi:10.3390/polym15173614)

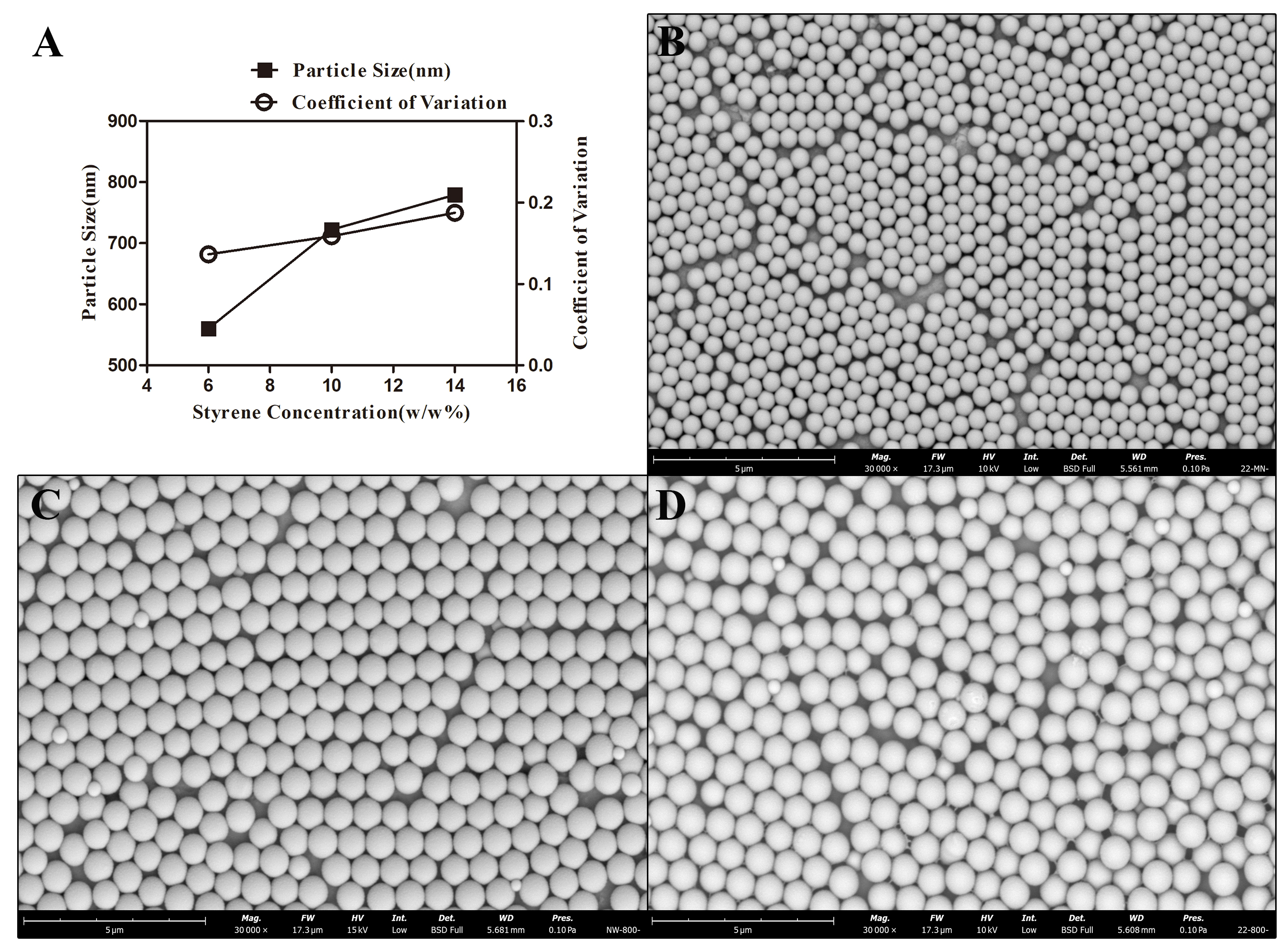

Supplement: Supplementary file 1 [file polymers-15-03614-s001.zip › FigureS1.jpg]

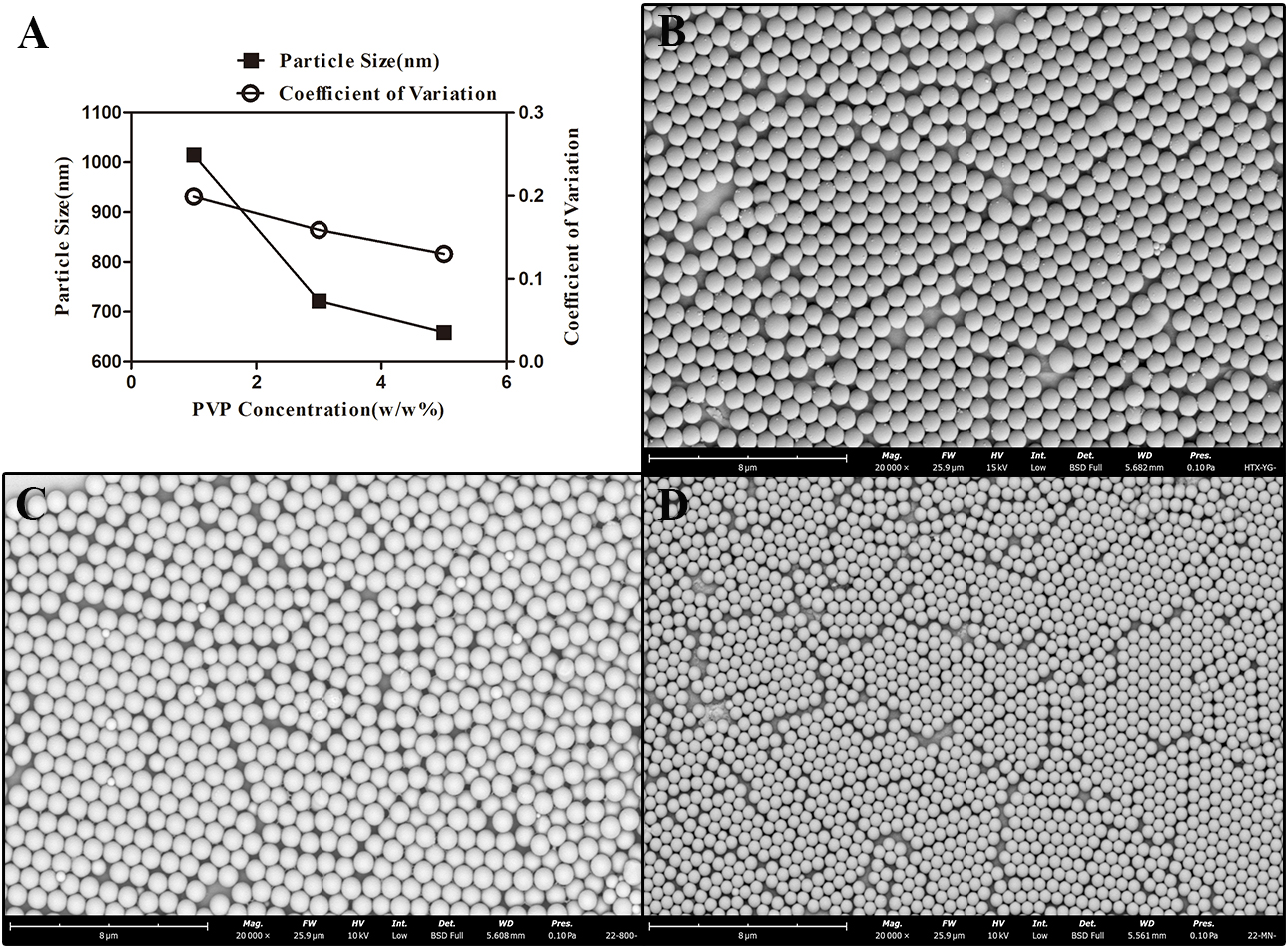

Supplement: Supplementary file 1 [file polymers-15-03614-s001.zip › FigureS2.jpg]

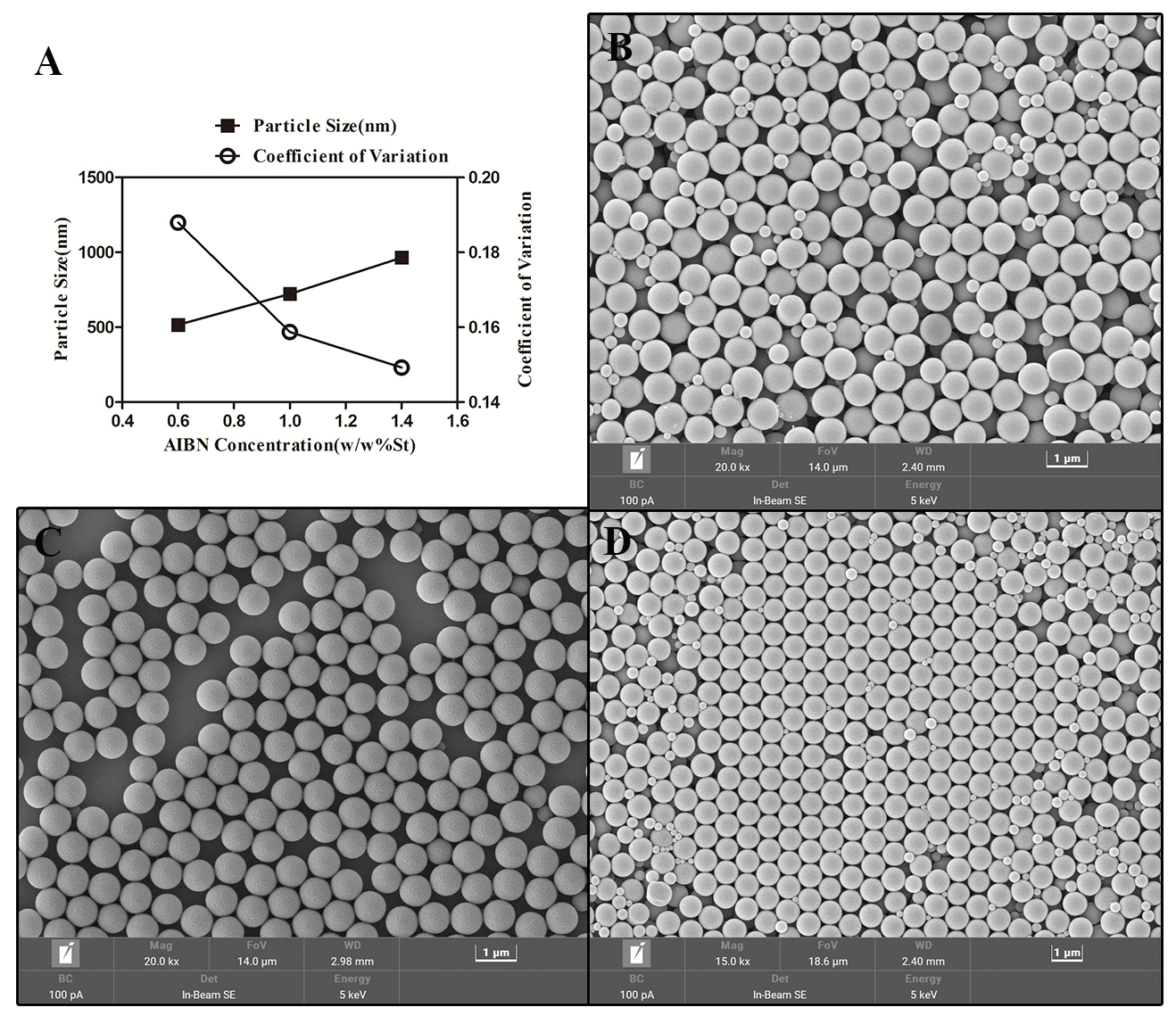

Supplement: Supplementary file 1 [file polymers-15-03614-s001.zip › FigureS3.jpg]

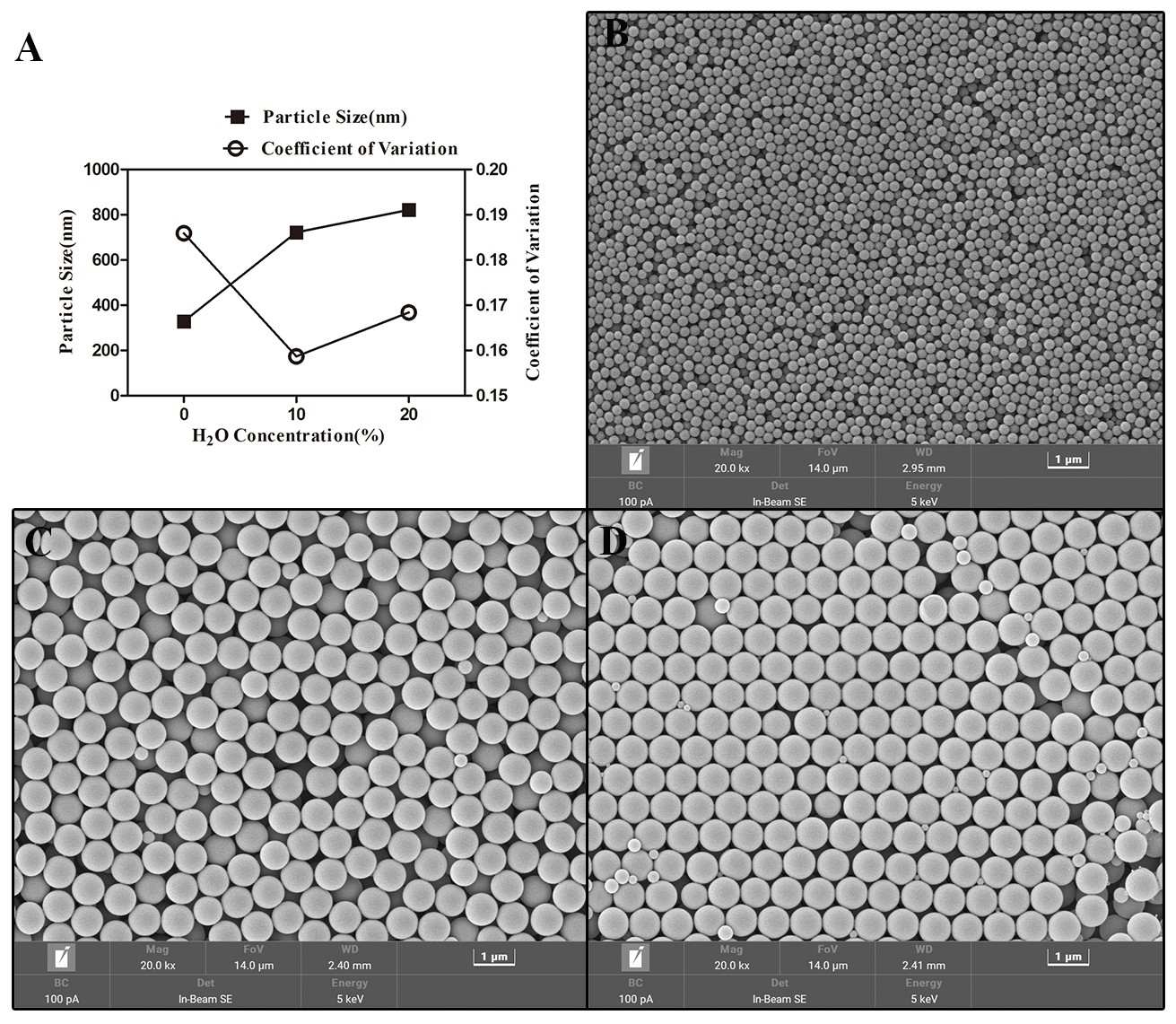

Supplement: Supplementary file 1 [file polymers-15-03614-s001.zip › FigureS4.jpg]

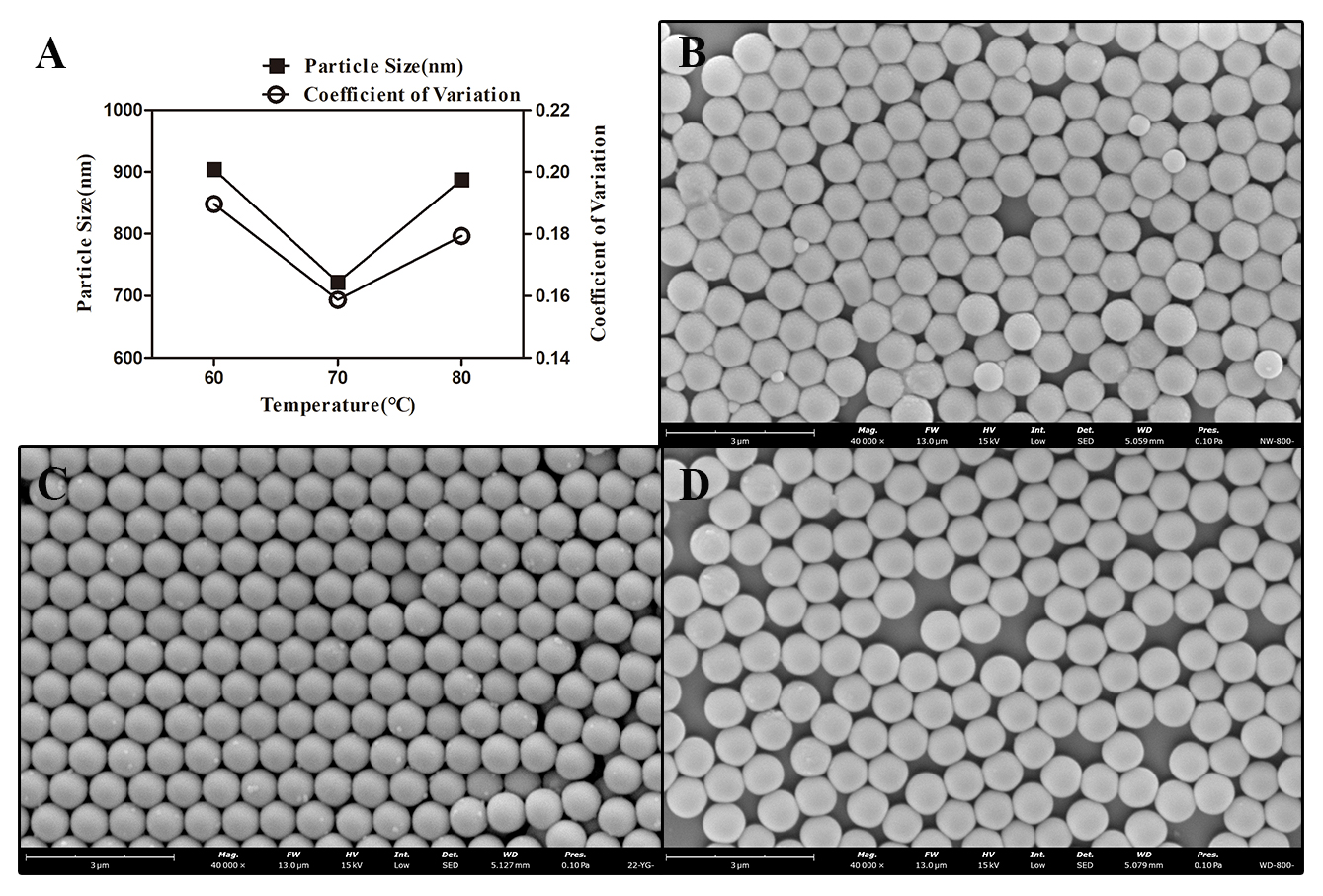

Supplement: Supplementary file 1 [file polymers-15-03614-s001.zip › FigureS5.jpg]
